# Hot topics in cellular neuropathology III: using CRISPR/Cas9 technology for deciphering central nervous system disease targets

**DOI:** 10.3389/fncel.2023.1276077

**Published:** 2023-09-01

**Authors:** Dirk M. Hermann

**Affiliations:** Department of Neurology, University Hospital Essen, University of Duisburg-Essen, Essen, Germany

**Keywords:** CRISPR/Cas9 technology, gene editing, gene therapy, neurodegenerative disease, neurodevelopment

Methodological advancements have played a pivotal role in driving progress within the field of biomedicine. A striking illustration of this trend is the groundbreaking discovery of the CRISPR/Cas9 technology by Emmanuelle Charpentier and Jennifer A. Doudna in 2012, a feat that earned them the Nobel Prize in Chemistry in 2020. This innovation, which facilitates precise gene editing, has revolutionized the capability to incorporate gene sequences into genomes with an unprecedented level of accuracy and efficiency (Jinek et al., 2012).

The genesis of the CRISPR/Cas9 technology emerged unexpectedly from investigations into *Streptococcus pyogenes*. Within this context, Charpentier uncovered an entirely novel molecule, *tracrRNA*, which constitutes a crucial component of the bacterium's innate defense mechanism aimed at neutralizing viruses by cleaving their DNA strands (Deltcheva et al., 2011). Through strategic manipulation of the bacterium's molecular components and the adaptation of its enzymatic machinery, Charpentier and Doudna ingeniously reprogrammed these molecular “scissors,” endowing them with the ability to cleave DNA molecules at predetermined locations (Jinek et al., 2012). This remarkable technique, when extended to eukaryotic cells, has unlocked unparalleled avenues in the realm of gene editing, thereby profoundly augmenting our comprehension of cellular physiology and pathology.

Consequently, the CRISPR/Cas9 technology has emerged as a cornerstone of modern biotechnology over the past decade. Its global utilization across bioscience domains underscores its impact and significance, and it applications have extended far into the field of clinical neurosciences. So far, CRISPR/Cas9 has inseminated Cellular Neuropathology in the following ways:

- Treating Genetic Disorders: CRISPR/Cas9 technology holds promise for treating genetic disorders in the nervous system. Researchers have been exploring its potential for correcting mutations associated with neurodegenerative diseases such as Huntington's disease (Morelli et al., 2023) and spinal muscular atrophy (SMA) (Zhou et al., 2018), and certain types of epilepsy (Colasante et al., 2020).
- Modeling Neurological Disorders: CRISPR/Cas9 has been used to generate cellular and animal models of various neurological disorders. This allows scientists to study disease mechanisms more accurately, potentially leading to insights into the underlying causes of disorders like Alzheimer's disease (Schrauben et al., 2020), Parkinson's disease (Yang et al., 2019), and autism (Elamin et al., 2023).
- Understanding Neurodevelopment: By precisely manipulating genes in developing organisms, researchers have been able to uncover the roles of specific genes in brain development (Zhang et al., 2018). This knowledge can shed light on conditions like intellectual disabilities and developmental disorders.
- Functional Genomics: CRISPR/Cas9 technology has enabled scientists to perform large-scale functional genomics studies in neural cells (Sandberg et al., 2018), identifying genes that play key roles in neural function and connectivity. This could provide valuable insights into conditions involving altered neural circuits.
- Pain Management: Researchers have explored the use of CRISPR/Cas9 technology to modulate pain perception by targeting specific genes involved in pain pathways (Reese et al., 2020). This could potentially lead to more effective and targeted pain management strategies.
- Gene Therapies for Neurological Disorders: CRISPR/Cas9-based gene therapies have shown promise in preclinical studies for various neurological disorders (Ou et al., 2020). These therapies involve editing or modifying genes in specific brain regions to treat conditions like epilepsy or neurodegenerative diseases.
- Regenerative Medicine: CRISPR/Cas9 technology has been investigated for promoting neuronal regeneration after CNS injuries (Keatinge et al., 2021). Targeted gene editing could potentially stimulate the growth and repair of damaged neural tissue.

It is important to note that while CRISPR/Cas9 technology holds immense potential, there are also challenges to overcome, including off-target effects, delivery methods, and the need for rigorous safety assessments before any clinical applications can be more widely adopted. The use of CRISPR/Cas9 in clinical neuroscience has also brought about important ethical discussions. Ensuring the safety and specificity of gene editing techniques in the complex and delicate nervous system is a critical consideration.

In an effort to identify the most promising concepts in translational neurosciences, the Cellular Neuropathology section of Frontiers in Cellular Neurosciences recently launched a platform, the Hot Topics hub (Hermann, 2020). Within this platform, this journal searches for impactful papers in Cellular Neuropathology, which carry landscape-changing potential, broaden imagination horizons and expand current diagnostic or therapeutic possibilities. Within this platform, two previous Research Topics evaluated the role of subtle neuroinflammation in chronic neurodegeneration (Hermann et al., 2022) and the roles of neuronal plasticity in the injured CNS (Hermann, 2022). Within this new Research Topic, we would like to shift the focus from distinct disease pathomechanisms to a methodological tool, which broadly impacts various disease areas. As in the previous Research Topics, suitable manuscripts should push our understanding of neurological diseases, overcome existing limitations, pave the way for therapeutic progress and deserve attention in future research developments. Papers studying disease targets are welcome in the central and peripheral nervous systems, similar as papers studying CRISPR/Cas9-based gene therapies *in vitro* and *in vivo*. With this Research Topic, we would like to expand current knowledge in the field functional genomics and gain further insight into neurodevelopmental disorders. This Research Topic is open for all disease areas. Papers outlining limitations and challenges of CRISPR/Cas9 technologies are particularly invited. In this search for the best ideas and concepts, Original research, Reviews, Perspectives and Opinions are envisaged. Papers will be reviewed based on excellence, originality and innovation potential. Outstanding papers will be featured in an editorial. We are looking forward to your contributions to this new Research Topic.

## Author contributions

DH: Conceptualization, Investigation, Writing—original draft, Writing—review and editing.
